# A Transdiagnostic Video-Based Internet Intervention (Uni Virtual Clinic-Lite) to Improve the Mental Health of University Students: Randomized Controlled Trial

**DOI:** 10.2196/53598

**Published:** 2024-08-13

**Authors:** Louise M Farrer, Hayley M Jackson, Amelia Gulliver, Alison L Calear, Liana Leach, Penelope Hasking, Natasha Katruss, Philip J Batterham

**Affiliations:** 1 Centre for Mental Health Research National Centre for Epidemiology and Population Health The Australian National University Canberra Australia; 2 Department of Health Economics Wellbeing and Society, National Centre for Epidemiology and Population Health, The Australian National University Canberra Australia; 3 Curtin enAble Institute and School of Population Health, Faculty of Health Sciences, Curtin University Curtin Australia

**Keywords:** university students, internet-based intervention, randomized controlled trial, mental health, transdiagnostic

## Abstract

**Background:**

Numerous studies have demonstrated the effectiveness of digital interventions for improving the mental health of university students. However, low rates of engagement with these interventions are an ongoing challenge and can compromise effectiveness. Brief, transdiagnostic, web-based video interventions are capable of targeting key mental health and related issues affecting university students and may be more engaging and accessible for this population.

**Objective:**

This study used a 2-arm randomized controlled trial to evaluate the effectiveness of Uni Virtual Clinic-Lite (UVC-Lite), a fully automated, transdiagnostic, web-based video intervention, relative to an attention-control condition. The primary outcomes were symptoms of depression and generalized anxiety disorder. The secondary outcomes included psychological distress, social anxiety symptoms, body appreciation, quality of life, well-being, functioning, general self-efficacy, academic self-efficacy, and help seeking. Program use (intervention uptake and engagement) and satisfaction were also assessed.

**Methods:**

University students (n=487) with mild to moderate symptoms of distress were recruited from universities across Australia and randomly allocated to receive access to the UVC-Lite intervention or an attention-control condition targeting general health for a period of 6 weeks. UVC-Lite includes 12 modules, each comprising a brief animated video and an accompanying exercise. Of the 12 modules, 7 also included a brief symptom screening quiz. Outcomes were assessed at baseline, postintervention, and 3- and 6-months postintervention.

**Results:**

The primary and secondary outcomes were analyzed on an intention-to-treat basis using mixed models repeated measures ANOVA. The intervention was not found to be effective relative to the control condition on any of the primary or secondary outcomes. While 67.9% (114/168) of participants accessed at least 1 module of the intervention, module completion was extremely low. Subgroup analyses among those who engaged with the program (completed at least 1 video) and those with higher baseline distress (Distress Questionnaire-5 score ≥15) did not reveal any differences between the conditions over time. However, uptake (accessing at least 1 video) and engagement (completing at least 1 video) were higher among those with higher baseline symptoms. Satisfaction with the intervention was high.

**Conclusions:**

The UVC-Lite intervention was not effective relative to a control program, although it was associated with high satisfaction among students and was not associated with symptom deterioration. Given the challenges faced by universities in meeting demand for mental health services, flexible and accessible interventions such as UVC-Lite have the potential to assist students to manage symptoms of mental health problems. However, low uptake and engagement (particularly among students with lower levels of symptomatology) are significant challenges that require further attention. Future studies should examine the effectiveness of the intervention in a more highly symptomatic sample, as well as implementation pathways to optimize effective engagement with the intervention.

**Trial Registration:**

Australian New Zealand Clinical Trials Registry ACTRN12621000375853; https://www.anzctr.org.au/Trial/Registration/TrialReview.aspx?id=380146

## Introduction

### Background

There is growing concern about the mental health of university students worldwide. Most students commence university study during the transition from late adolescence to early adulthood, a time when the first onset of many common mental disorders is high [1,2]*.* In addition, this group faces numerous stressors such as transition to independent living, instability in relationships, financial stress, and academic pressure [3-6]; these stressors increase their risk of developing mental health problems [7]. Moreover, the COVID-19 pandemic and the associated mitigation measures severely disrupted the daily lives and educational experiences of university students around the world [8], with students reporting increased academic challenges due to a shift to remote learning, disruptions to paid work, concerns about their future academic and career prospects, and increased social isolation due to campus closures and physical distancing mandates [8-11].

Before the COVID-19 pandemic, research indicated that approximately one-third of university students met criteria for at least 1 mental disorder during a 12-month period, with generalized anxiety and mood disorders being the most prevalent diagnoses [12,13]. Furthermore, studies of the prevalence of psychological distress among university student populations have indicated that 19% to 48% of students experience high levels of psychological distress and that as many as 65% experience subsyndromal symptoms indicative of mild to moderate mental illness [14,15]. Research conducted since the onset of the pandemic has demonstrated an increase in the prevalence of mental health problems among university students [11,16,17], and approximately 30% to 40% of students are estimated to have experienced symptoms of depression or anxiety during the first 2 years of the pandemic [18-20]*.*

Untreated mental disorders in young people place them at higher risk for developing comorbid mental disorders in later adulthood [21,22]. Moreover, mental health problems among university students can negatively impact academic performance [23] and have been associated with an increased risk of dropping out of university [24]. However, only approximately one-sixth of university students with a mental disorder receive minimally adequate treatment [12]. The most frequently reported barriers to using mental health services by students include a preference for self-management, concerns about stigma, high treatment costs, and scheduling difficulties [25]. Moreover, traditional models of delivering psychotherapy (ie, individual face-to-face sessions) are unlikely to be sufficient to meet the growing demand for services in universities and may not be feasible during times of crisis (such as during the COVID-19 pandemic). Therefore, there is an urgent need to reimagine existing models of mental health service delivery in tertiary education settings.

Internet-based and other digital mental health interventions are proposed as one solution to increase access to evidence-based treatment and prevention among the university student population. University students have indicated a willingness to use digital programs [26,27] and perceive numerous advantages in doing so, including increased treatment accessibility, anonymity, privacy, and confidentiality, as well as the potential to mitigate financial concerns by accessing support at a lower cost [28]. Moreover, several systematic reviews and meta-analyses have demonstrated that digital mental health interventions can be effective for university students [29-33]. In particular, these reviews have shown that interventions based on cognitive behavioral therapy (CBT) have been the most frequently used therapeutic approaches and demonstrate larger effects than other approaches [32]. However, the effectiveness of current interventions is also limited by low levels of adherence and high rates of dropouts [34].

Due to high rates of comorbidity between mental disorders in university student populations [35], transdiagnostic approaches that deliver therapeutic content targeting the common mechanisms that underlie mental disorders may have more clinical utility than disorder-specific approaches. Internet-based programs targeting the reduction of depression and anxiety symptoms using transdiagnostic approaches have generally been shown to be effective in improving one or more mental health outcomes among university students relative to control groups [36-38] although there are exceptions to this [7,39]. However, the potential of transdiagnostic internet-based interventions in university student populations has not yet been fully realized. Recent trials of transdiagnostic internet-based interventions with university students have predominantly focused on interventions delivered with guidance from a health professional or trained nonprofessional [7,40]. Although the provision of therapeutic guidance in an intervention can have small positive effects on the rates of intervention completion [38], this also requires increased resources, which substantially reduces the potential for scalability [41]. Moreover, sustained and active engagement with existing transdiagnostic interventions may be challenging for university students for different reasons. Those with few symptoms may not have much motivation for change or may not see a need to engage. Those who experience mental ill health or struggle to balance competing demands may find it challenging to engage with complex interventions that contain many pages of information; are predominantly text based; or include multiple components (eg, written text, audio and visual information, and exercises and worksheets) [42]. In addition, most previous randomized controlled trials have tested transdiagnostic interventions using relatively small sample sizes (eg, ≤100 participants), which have not been sufficiently powered to detect small, yet clinically meaningful, effects in mental health outcomes that may be present in this population [7].

There is a need to explore innovative methods of delivering transdiagnostic programs that are low intensity and use potentially more engaging methods (eg, videos) to deliver therapeutic content directly to the user. A previous trial of a brief video-based transdiagnostic intervention in a sample of adults from the general population with elevated levels of psychological distress demonstrated significant reductions in depression, panic, and social anxiety symptoms relative to an attention-control condition [43]. Video-based interventions may be particularly accessible for university students who have limited time or motivation, as videos require less reading, scrolling, or shifting between pages of text-based materials or different intervention components. A recent study found that university students show a preference for internet-based mental health interventions that contain video content [44]. However, there are currently no video-based internet interventions that have been developed for and trialed with the university student population. In recognition of the potential utility of a video-based transdiagnostic internet intervention for university students, our research group developed the Uni Virtual Clinic-Lite (UVC-Lite), a brief intervention designed to help university students manage mental health problems and related issues.

### Objective

In this 2-arm randomized controlled trial, we compared the unguided use of the UVC-Lite intervention for 6 weeks with an attention-control condition among students recruited from universities across Australia. The primary aim of the trial was to assess the feasibility and effectiveness of the intervention in reducing the symptoms of depression and generalized anxiety disorder at immediate postintervention and longer term at 3- and 6-month follow-ups. A range of secondary outcomes, as well as satisfaction with and use of the intervention, were also examined.

## Methods

### Participants (Recruitment and Eligibility Criteria)

Undergraduate and postgraduate students from all 42 universities in Australia were targeted using the recruitment strategies outlined below. Recruitment took place between August 2021 and May 2022, and all the follow-up data were collected by January 2023. Multiple strategies were used to recruit students into the trial. Students were provided with information about the trial via targeted Instagram and Facebook advertisements; via posts to university-affiliated groups on social media sites (Facebook, Instagram, Reddit, and Discord); and through university media channels such as newsletters. Recruitment materials targeted students interested in learning more about mental health and well-being. Student advocacy departments and associations, student housing coordinators, marketing teams, survey management departments, university counselors and well-being staff, university psychology clinics, and academic course conveners were contacted via email and social media pages to request assistance in distributing information about the trial to students via emails and flyers. Individuals involved in distributing recruitment materials were not asked to target any specific student groups.

To be eligible for the trial, students were required to (1) be enrolled at an Australian university; (2) currently reside in Australia; (3) be aged between 18 and 25 years; and (4) score between 8 and 17 on the Distress Questionnaire-5 (DQ5), which indicates moderate levels of psychological distress [45]. UVC-Lite was designed to be a brief internet-based self-help intervention; thus, students with a moderate level of psychological distress were targeted, as these programs are thought to be most suitable for this group [46]. However, because of the concerns noted in the ethical review about offering this program to people with higher levels of distress, students with high psychological distress (DQ5 score of 18-25) were deemed ineligible and were instead provided with specific instructions on how to access resources for help seeking. Students with a diagnosis of bipolar disorder, schizophrenia, posttraumatic stress disorder, or a personality disorder could participate if they fulfilled the eligibility criteria and were also currently receiving support or treatment for their disorder.

### Procedure

Students interested in participating in the trial were provided with a link to a Qualtrics survey (Qualtrics International Inc) containing the participant information sheet, a question to obtain informed consent, and self-report questions to determine trial eligibility. Eligible participants were asked to provide an email address and complete the baseline survey. Participants who completed the baseline survey were randomized to either the UVC-Lite intervention condition or an attention-control condition for a period of 6 weeks. An independent researcher who was not involved with the trial generated a random sequence of integers between the values of 1 and 2 using a web-based application [47] and manually allocated participants to the trial conditions according to this sequence to ensure allocation concealment. Trial allocations were sent to a research assistant (NK), who enrolled participants in the trial.

During the 6-week intervention period, participants were sent 2 automated emails per week containing a link to an intervention or control module hosted in Qualtrics software*.* Participants could not access modules until they received these emails. Emails contained information about the topic of each module. Participants were not restricted in their use of usual services or support during the intervention or follow-up periods. All participants received an email containing a link to the postintervention survey 6 weeks after completing the baseline survey, and links to the follow-up surveys were sent 3 and 6 months after the postintervention survey was distributed. Participants were sent 2 email reminders to complete the postintervention and follow-up surveys. Participants who completed all the assessments were entered into a prize draw to win one of ten AUD $100 (USD $67) electronic gift cards. In case of distress, participants could contact the research team and be followed up by the primary researcher (LMF), a registered psychologist, and provided with support and referral to relevant health, counseling, and crisis support services. No students in the trial contacted the research team in distress.

### Ethics Approval

The trial was registered at the Australian New Zealand Clinical Trials Registry (ACTRN12621000375853), and ethical approval for the study was obtained from the Australian National University Human Research Ethics Committee (protocol #2020/412).

### Intervention Condition: UVC-Lite

UVC-Lite is a brief internet-based intervention that comprises 12 modules targeting the common mechanisms that underlie mental health problems in university students (Multimedia Appendix 1). Each module includes a 3- to 6-minute video in which a series of animated characters introduce and share their lived experience of one or more mental health problems or issues affecting mental health in students. A therapist character presents relevant psychoeducation and therapeutic techniques, and a downloadable worksheet exercise designed to facilitate the practice of the therapeutic techniques presented follows each video. Additional features include self-monitoring quizzes for participants to track and receive feedback on their symptoms (included in modules 1, 3, 4, 5, 7, and 11) and links to additional help-seeking resources provided at their university or in the community if participants score above a certain symptom severity cutoff. Module transcripts and exercises were available for participants to download. The 12 modules covered the following topics and therapeutic strategies and were presented to participants in this order: (1) dealing with depression and low mood (behavioral activation: pleasant events scheduling); (2) tackling negative and anxious thoughts (cognitive reframing: thought diary), (3) dealing with anxiety (cognitive reframing, breathing, and grounding techniques); (4) managing study issues: procrastination and time management (practical strategies for time management); (5) perfectionism (challenging perfectionistic thoughts and behaviors); (6) coping with stress (mindfulness practice: focus on the present moment and mindful eating activity); (7) managing sleep issues (sleep hygiene); (8) social anxiety and shyness (behavioral experiments or exposure techniques); (9) relationships and loneliness (communication skills and social support); (10) social media use (practical strategies for reducing social media use); (11) body image (body functionality appreciation exercise); and (12) thoughts of suicide (safety planning exercise).

The content for the UVC-Lite program was drawn from a previously developed internet-based mental health intervention for university students, the Uni Virtual Clinic. Although this intervention was not effective in reducing the symptoms of depression, anxiety, or distress compared with a waitlist control group in a previous trial, it was shown to be effective in reducing the symptoms of social anxiety and improving academic self-efficacy [43]. The original Uni Virtual Clinic program, which was developed using extensive co-design processes involving students, university teaching and administrative staff, and service providers, was designed to be a comprehensive portal for student mental health [48]. We developed the UVC-Lite program based on the feedback from users of the original Uni Virtual Clinic program to ensure that it was focused primarily on the most critical issues affecting student mental health. The UVC-Lite incorporates key elements from the original program but is delivered in a more concise format involving brief videos containing student stories, tailored psychoeducation, and a range of psychotherapeutic strategies.

### Attention-Control Condition: General Health Information

The attention-control condition received biweekly emails containing a link to the information pertaining to general health rather than mental health. The health information was delivered in a PDF document accessed through a weblink and was approximately matched to the UVC-Lite program on completion time. A similar program has previously been shown not to be associated with therapeutic reductions in depression [49]. A total of 12 general health topics were covered, including bone health, sun exposure, food hygiene, dietary supplements, kidney health, microbes, household burns, respiratory viruses, heart health, allergens, posture, and pancreas health. The general health information was derived from web-based public domain articles about general health topics published by the National Institutes of Health [50].

### Measures

#### Overview

At baseline, the following demographic and study data were collected: age, sex, ethnicity, languages spoken, country of birth, living situation, state or territory of residence, hours in paid employment, financial stress, relationship status, study discipline and year of degree, study load, international or domestic student status, academic performance, and engagement with student life. Primary and secondary outcomes (described in subsequent sections) were assessed at the baseline, postintervention time point, and 3- and 6-month follow-ups. Satisfaction with the intervention program was assessed at the postintervention time point.

#### Primary Outcomes

Primary outcomes were symptoms of depression (measured by the Patient Heath Questionnaire-9 [PHQ-9]) [51] and generalized anxiety (measured by the Generalized Anxiety Disorder Scale-7 [GAD-7]) [52]. The PHQ-9 comprises 9 items rated on a 4-point scale, ranging from “not at all” to “nearly every day.” Item scores are summed to produce an overall severity score ranging from 0 to 27, with higher scores indicating greater symptom severity (0-4=no symptoms, 5-9=mild symptoms, 10-14=moderate symptoms, and 15-27=severe symptoms). The GAD-7 comprises 7 items rated on the same 4-point scale as the PHQ-9. Summed scores produce an overall severity score ranging from 0 to 21 (0-4=no symptoms, 5-9=mild symptoms, 10-14=moderate symptoms, and 15-21=severe symptoms). Higher scores indicate greater symptom severity. Both scales have been shown to have robust psychometric properties in general population samples and detect change over time [53,54]. In the current sample, internal consistency was acceptable at baseline (PHQ-9: α=.80; GAD-7: α=.84).

#### Secondary Outcomes

The secondary outcomes included social anxiety symptoms, psychological distress, body appreciation, quality of life, well-being, functioning, general self-efficacy, academic self-efficacy, and help-seeking behavior.

#### Social Anxiety

Symptoms of social anxiety were assessed using the Social Anxiety Disorder Screener [55], a brief 4-item scale with symptom severity scores ranging from 0 to 16. This scale has been developed and validated using Australian community–based samples, has demonstrated good convergent and divergent validity with diagnostic measures [55], and had very good internal consistency in this sample (α=.90).

#### Psychological Distress

Psychological distress was measured using the DQ5 [45], which consists of 5 items rated on a 5-point scale that assess the symptoms of common mental disorders experienced over the last 30 days (range 5-25; 5-7-mild distress, 8-17=moderate distress, and 18-25=high distress). The DQ5 has been shown to accurately identify individuals at risk for specific mental disorders relative to the Kessler-6 and Kessler-10 screeners [45]. Previous studies have demonstrated that DQ5 displays high internal consistency and external validity [45,56]. Internal consistency in the current sample was α=.58; however, this is an artifact of the restricted range on the DQ5 of the recruited sample [57,58].

#### Body Appreciation

Body appreciation was assessed using the 10-item Body Appreciation Scale-2 (BAS-2) [59]. The BAS-2 comprises 10 items rated on a 5-point scale. Item responses are averaged to produce a total score ranging from 1 to 5, with higher scores indicating greater body appreciation. The BAS-2 has demonstrated good internal consistency, test-retest reliability, and construct validity [59]. The scale had good internal consistency in this sample (α=.95).

#### Quality of Life

Quality of life was assessed using the European Health Interview Survey-8 (EUROHIS-8), an 8-item measure designed to assess the psychological, physical, social, and environmental aspects of quality of life (range 0-32) [60]. The EURO-HIS 8 has satisfactory discriminant validity and acceptable internal consistency [61], and internal consistency was adequate in the current sample (α=.79).

#### Well-Being

Well-being was assessed using the 5-item World Health Organization Well-Being Index, a 5-item scale with item responses ranging from 0 to 6, which produces a raw well-being score ranging from 0 to 25. Raw scores are multiplied by 4 to provide a final score ranging from 0 (worst-imaginable well-being) to 100 (best-imaginable well-being) [62]. The 5-item World Health Organization Well-Being Index has demonstrated sensitivity and specificity in screening for depression and is sensitive to change in clinical trials [63]. The internal consistency of the scale in the current sample was adequate (α=.82).

#### Functioning

Functioning was measured by the Recovering Quality of Life scale, a 20-item measure assessing the dimensions of mental health functioning including activity, hope, belonging and relationships, self-perception, well-being, autonomy, and physical health. The items are rated on a 5-point scale ranging from 0 to 4, with total scores ranging from 0 to 80. The scale has acceptable internal consistency, test-retest reliability, and convergence with related measures [64]. Internal consistency in the current sample was good (α=.88).

#### General Self-Efficacy

Self-efficacy was measured using the 10-item General Self-Efficacy Scale, which measures perceived coping with daily hassles and adaptation to stressful life events on a 4-point scale (range 0-30) [65]. The 10-item General Self-Efficacy Scale has been validated in 31 countries and languages and has acceptable internal consistency and good discriminant and concurrent validity [66,67]. Internal consistency in this study was good (α=.88).

#### Academic Self-Efficacy

Academic self-efficacy (ie, confidence in one’s ability to successfully complete university-related tasks) was assessed using the Study and Social subscales of the College Self-Efficacy Inventory (CSEI; 15 items, 5-point Likert scale ranging from very unconfident to very confident, range 0-60) [68]. The CSEI Course subscale measures self-efficacy to complete tasks associated with university study (eg, “How confident do you feel to keep up to date with your university work?”). The CSEI Social subscale measures self-efficacy related to social interactions at university (eg, “How confident do you feel to talk to your lecturers/tutors?”). Both subscales have demonstrated adequate internal consistency (α=.79-.86) and have been found to be negatively correlated with social anxiety and academic worry or concern and positively correlated with grade point average [69,70]. Internal consistency in this study was good (α=.87).

#### Help Seeking

Help seeking was measured with the Actual Help Seeking Questionnaire [71]. Participants were asked whether they had sought help from a range of formal (ie, general practitioner, psychologist, counselor, and helpline) and informal (ie, friends, family, and partner) help sources in the past month. The Actual Help Seeking Questionnaire has been found to adequately differentiate help-seeking behavior for different problems and help sources [71].

#### Intervention Satisfaction and Adherence

Program use was assessed using author-developed JavaScript embedded within Qualtrics to track clicks and percentage of videos watched. Measures included uptake (number of modules and videos started) and engagement (viewing at least 50% of each video, number of exercises accessed, and number of quizzes completed). Satisfaction was assessed by asking participants how satisfied they were with the intervention (5-point scale, *very unsatisfied* to *very satisfied*); whether they would recommend the intervention to other students (4-point scale, *definitely not* to *yes, definitely*); how much they learned from the intervention (4-point scale, from *almost nothing* to *a great deal*); and which modules they liked the best.

### Blinding

Trial researchers were blinded to treatment allocation. Participants were blinded to whether they received the active or attention-control intervention. Specifically, participants were informed that they would be randomized to receive 1 of 2 programs: information and strategies for improving mental health (UVC-Lite) or information and strategies for improving general health (attention control). They were not provided with information about which program was expected to be more effective. Assessments were also blinded, as self-report questionnaires were delivered on the web.

### Statistical Analyses

Statistical analyses were conducted by the primary author (LMF) and author PJB using SPSS (version 28; IBM Corp) for Windows [72]. Sample characteristics were described at baseline, and differences between the intervention and control groups were examined using 2-tailed *t* tests and chi-square statistics. Predictors of dropout (failure to complete trial assessments) were examined at postintervention and at 3- and 6-month follow-ups using logistic regression. Predictors of uptake and engagement with the UVC-Lite intervention were also examined using logistic regression. The primary and secondary outcome variables were analyzed on an intention-to-treat basis using mixed models repeated measures ANOVA, with measurement occasion as a within-groups factor and trial condition as a between-groups factor [73]. Mixed models incorporate all available data under the assumption that data are missing at random. Within-person variation was modeled using an unstructured covariance matrix. To examine whether intervention effectiveness was related to intervention adherence or baseline symptoms, analyses were re-estimated among participants who watched at least 1 video and those with higher levels of baseline distress (DQ5 score ≥15). Logistic regression was used to examine the relationship between trial condition and help-seeking behavior at postintervention and at 3- and 6-month follow-ups.

### Power

A power calculation based on repeated measures ANOVA (conducted using G*Power) indicated that a target sample size of 418 would enable the detection of an effect of Cohen *d*=0.3 between conditions, with 90% power and a significance level of α=.01. The recruited sample size was 487, allowing the detection of an effect of Cohen *d*=0.3 at a significance level of .01 with slightly higher power (~95%). However, high attrition was observed in the trial, with a sample of 265 retained at postintervention, 105 at 3 months, and 172 at 6 months.

## Results

### Trial Flow and Survey Completion

**Figure 1:**
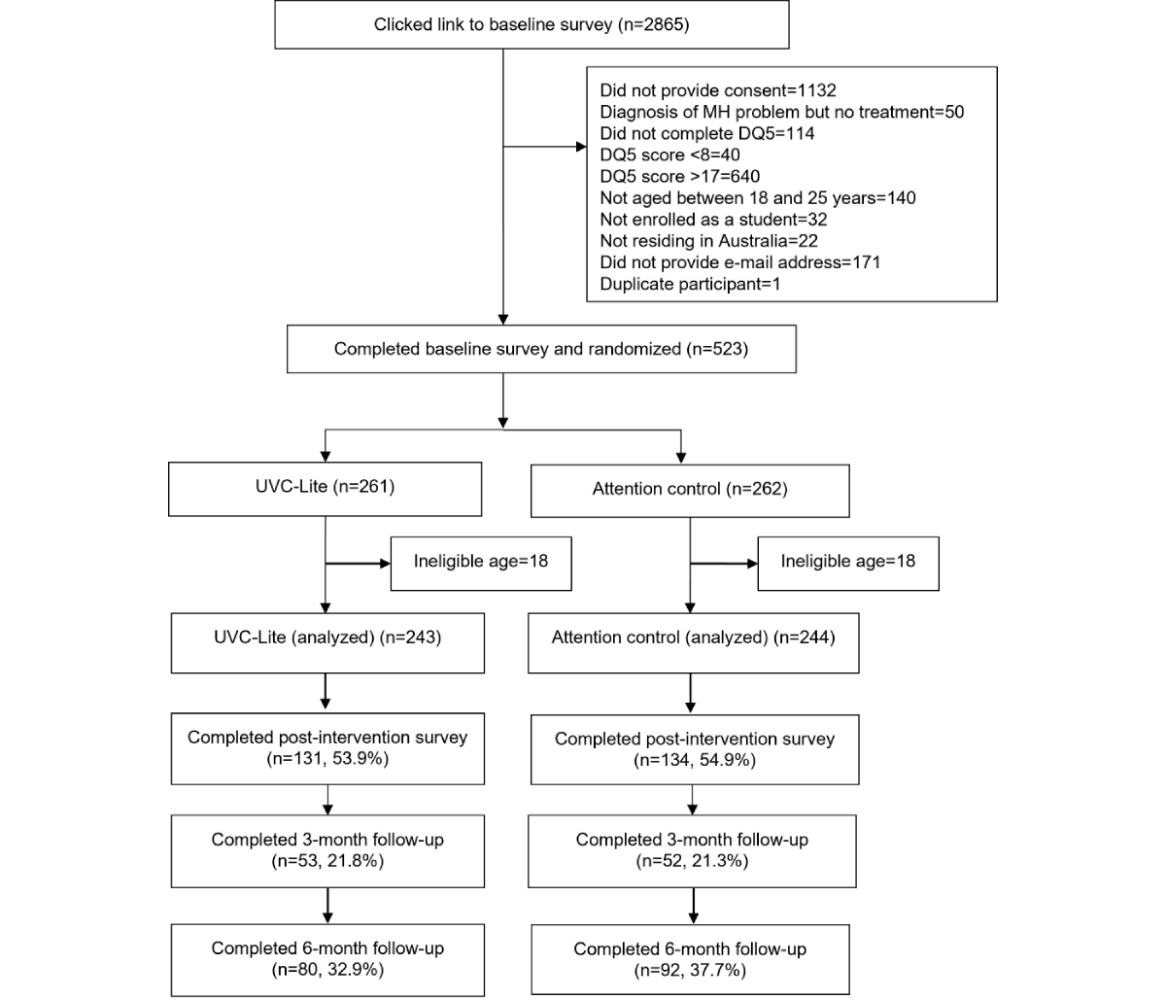
Trial flow. DQ-5: Distress Questionnaire; MH: mental health; UVC-Lite: Uni Virtual Clinic-Lite.

The flow of participants through the trial is shown in Figure 1. A total of 2865 individuals clicked on the study invitation. Of them, 523 (18.25%) completed the baseline survey and were randomized into trial conditions. The most common reasons for not progressing to randomization were clicking on the link but not reading the study information and providing consent (n=1132, 39.5%) and scoring >17 on the DQ5 (n=640, 22.3%). After completing the baseline survey, 36 participants were identified as being outside the eligible age range for the trial and were excluded from analyses, leaving 487 eligible participants. A total of 265 participants (54.4%) completed the postintervention survey, 105 (21.6%) completed the 3-month follow-up survey, and 172 (35.3%) completed the 6-month follow-up survey. In total, 2 errors were discovered during the trial that resulted in a delay in participants receiving either their condition allocation after completing the baseline survey or their 3-month follow-up survey. As a result of administrative errors, 129 (26.5%) participants experienced a delay of >3 days between completing their baseline assessment and receiving confirmation of their allocated trial condition and instructions for accessing their materials. A technical error with the survey delivery platform resulted in 188 (38.6%) participants not receiving their 3-month follow-up survey on the date when it was due to be administered. Baseline depression and generalized anxiety symptoms, trial condition, and whether trial condition allocation or 3-month follow-up survey was administered on time were examined as predictors of survey completion at postintervention and at 3-and 6-month follow-ups. Baseline symptoms and trial condition were unrelated to survey completion at all time points. However, delay in the administration of the 3-month follow-up survey due to technical error was significantly associated with increased odds of survey completion at 3 months (B=2.61; *P*<.001).

### Sample Characteristics

Table 1 presents the baseline demographic and clinical characteristics of the sample. Students from 40 institutions in Australia participated in the study. The sample (n=487) was predominantly female (n=355, 72.9%), and participants were aged on average 21 (SD 2.1) years. Most participants were domestic students (411/487, 84.4%) studying full time (449/487, 92.2%) at the undergraduate level (416/485, 85.8%). Participants were studying degrees from a range of different disciplines. Most students were in some form of paid employment (328/487, 67.4%) and were living either with family (209/487, 42.9%) or on campus (152/487, 31.2%). There were no differences between participants randomized to the intervention or control conditions on any of the baseline demographic or symptom variables. As expected, due to screening, distress scores were moderate at baseline, whereas baseline depression and generalized anxiety symptoms were within the mild range.

**Table 1:** Demographic and study characteristics of the sample.

				Intervention (n=243)		Control (n=244)		Total (n=487)		Chi-square (*df*)			*t* test (*df*)			*P* value	
**Categorical variables, n (%)**																	
	**Sex**										0.4 (2)			—^a^			.82
		Female	180 (74.1)		175 (71.7)		355 (72.9)										
		Male	52 (21.4)		58 (23.8)		110 (22.6)										
		Other	11 (4.5)		11 (4.5)		22 (4.5)										
	**Ethnicity**										3.2 (4)			—			.53
		Aboriginal and/or Torres Strait Islander and/or Pacific Islander	6 (2.5)		2 (0.8)		8 (1.6)										
		African/Middle Eastern	6 (2.5)		6 (2.5)		12 (2.5)										
		Asian/Indian	65 (26.7)		75 (30.7)		140 (28.7)										
		White/European	154 (63.4)		152 (62.3)		306 (62.8)										
		Other	11 (4.5)		8 (3.3)		19 (3.9)										
	**Current living situation**										4.6 (6)			—			.59
		With parents/family	100 (41.2)		109 (44.7)		209 (42.9)										
		On-campus housing	82 (33.7)		70 (28.7)		152 (31.2)										
		Friends off-campus	24 (9.9)		21 (8.6)		45 (9.2)										
		Others off-campus	19 (7.8)		18 (7.4)		37 (7.6)										
		With partner/children	10 (4.1)		17 (7)		27 (5.5)										
		Alone	7 (2.9)		9 (3.7)		16 (3.3)										
		Other	1 (0.4)		0 (0)		1 (0.2)										
	**Hours per week in paid employment**										6.05 (5)			—			.30
		None	89 (36.6)		70 (28.7)		159 (32.6)										
		1-9	53 (21.8)		58 (23.8)		111 (22.8)										
		10-19	68 (28)		67 (27.5)		135 (27.7)										
		20-29	24 (9.9)		33 (13.5)		57 (11.7)										
		30-39	8 (3.3)		15 (6.1)		23 (4.7)										
		≥40	1 (0.4)		1 (0.4)		2 (0.4)										
	**Relationship status**										2.50 (3)			—			.48
		Single	170 (70)		165 (67.6)		335 (68.8)										
		In relationship (not living together)	51 (21)		57 (23.4)		108 (22.2)										
		In relationship (living together)	20 (8.2)		22 (9)		42 (8.6)										
		Other	2 (0.8)		0 (0)		2 (0.4)										
	**Discipline of degree studied (n=438)**										5.46 (7)			—			.60
		Health/medicine	83 (38.4)		71 (32)		154 (35.2)										
		Arts/social sciences	37 (17.1)		42 (18.9)		79 (18)										
		Science	39 (18.1)		45 (20.3)		84 (19.2)										
		Engineering/computing	21 (9.7)		28 (12.6)		49 (11.2)										
		Business/economics	11 (5.1)		14 (6.3)		25 (5.7)										
		Law/criminology	14 (6.5)		15 (6.8)		29 (6.6)										
		Education	11 (5.1)		6 (2.7)		17 (3.9)										
		Tertiary preparation course	0 (0)		1 (0.5)		1 (0.2)										
	**Year of degree**										4.30 (3)			—			.23
		First-year undergraduate	93 (38.3)		86 (35.2)		179 (36.8)										
		Later-year undergraduate	107 (44)		112 (45.9)		219 (45)										
		Honors	5 (2.1)		13 (5.3)		18 (3.7)										
		Postgraduate	37 (15.2)		32 (13.1)		69 (14.2)										
	**Study load**										0.44 (1)			—			.51
		Full time	226 (93)		223 (91.4)		449 (92.2)										
		Part time	17 (7)		21 (8.6)		38 (7.8)										
	**Student status**										0.07 (1)			—			.79
		Domestic	204 (84)		207 (84.8)		411 (84.4)										
		International	39 (16)		37 (15.2)		76 (15.6)										
	**Average mark/grade achieved last semester (n=352)**										0.86 (4)			—			.93
		High distinction	41 (23.4)		44 (24.9)		85 (24.1)										
		Distinction	79 (45.1)		76 (42.9)		155 (44)										
		Credit	42 (24)		47 (26.6)		89 (25.3)										
		Pass	12 (6.9)		9 (5.1)		21 (6)										
		Fail	1 (0.6)		1 (0.6)		2 (0.6)										
	**Engagement with university life**										1.40 (4)			—			.84
		Not at all (only attend classes)	60 (24.7)		53 (21.7)		113 (23.2)										
		Somewhat	80 (32.9)		80 (32.8)		160 (32.9)										
		Moderate (participate in some university-based activities)	53 (21.8)		57 (23.4)		110 (22.6)										
		High	29 (11.9)		27 (11.1)		56 (11.5)										
		Extremely high (involved in student leadership activities within university)	21 (8.6)		27 (11.1)		48 (9.9)										
**Continuous variables** **, mean (SD)**																	
	Age (y)			20.67 (2.15)		20.57 (2.09)		20.62 (2.12)		—			0.55 (485)			.58	
	Depression (PHQ-9^b^)			9.70 (5.11)		9.02 (4.34)		9.36 (4.75)		—			1.59 (485)			.11	
	Generalized anxiety (GAD-7^c^)			7.84 (4.53)		7.28 (4.13)		7.56 (4.34)		—			1.41 (485)			.16	
	Social anxiety (SAD^d^)			5.91 (3.69)		6.29 (3.89)		6.10 (3.79)		—			–1.12 (485)			.26	
	Psychological distress (DQ5^e^)			14.10 (2.62)		13.92 (2.56)		14.01 (2.59)		—			0.77 (485)			.44	
	Body appreciation (BAS-2^f^)			3.40 (0.90)		3.35 (0.87)		3.38 (0.88)		—			0.73 (485)			.47	
	Quality of life (EURO-HIS-8^g^)			28.63 (5.16)		28.51 (4.87)		28.57 (5.01)		—			0.27 (485)			.79	
	Well-being (WHO-5^h^)			47.00 (18.43)		47.57 (17.39)		47.28 (17.90)		—			–0.35 (484)			.72	
	Functioning (ReQoL^i^)			45.24 (11.43)		45.64 (11.02)		45.44 (11.22)		—			–0.39 (484)			.70	
	Self-efficacy (GSE-10^j^)			19.14 (5.21)		18.87 (4.64)		19.01 (4.93)		—			0.61 (484)			.54	
	Academic self-efficacy (CSEI^k^)			34.53 (10.86)		33.97 (10.17)		34.25 (10.51)		—			0.59 (484)			.55	

^a^Not applicable.

^b^PHQ-9: Patient Health Questionnaire-9.

^c^GAD-7: Generalized Anxiety Disorder Scale-7.

^d^SAD: Social Anxiety Disorder Screener.

^e^DQ5: Distress Questionnaire-5.

^f^BAS-2: Body Appreciation Scale-2.

^g^EURO-HIS-8: European Health Interview Surveys-8.

^h^WHO-5: 5-item World Health Organization Well-Being Index.

^i^ReQoL: Recovering Quality of Life.

^j^GSE-10: General Self-Efficacy Scale-10.

^k^CSEI: College Self-Efficacy Inventory.

### Intervention Adherence and Use

Uptake of and engagement with the UVC-Lite intervention was low (Table 2). On average, participants (n=243) started (eg, clicked on the link directing them to the intervention) 5.1 (SD 5.08) of the 12 intervention modules, with 168 (69.1%) participants starting at least 1 module, and 51 (21%) participants starting all 12 modules. Among those who clicked through to the intervention modules, participants started an average of 3.58 (SD 4.23) of the 12 videos, with 114 (67.9%) participants starting at least 1 of the 12 videos and only 14 (8.3%) participants starting all 12 videos. Only 11 (6.5%) participants watched all 12 videos. Encouragingly, among those who started 1 or more videos, 92.6% of participants watched over half of the video once they started (on average). Engagement with the module exercises was lower than the completion of the quizzes. Participants accessed an average of 1.89 (SD 3.02) of the 12 module exercises, with 86 (51.2%) participants accessing 1 or more exercises, and only 1 (0.6%) participant accessing all 12. In contrast, participants completed an average of 4.14 (SD 2.73) of 7 quizzes, with 151 (89.9%) participants completing at least 1 quiz and 57 (33.9%) participants completing all 7. Age, gender, student status (domestic vs international), baseline distress (DQ5 score: 8-14 vs 15-17), receiving trial allocation information on time, and year of study (first year vs later year) were examined as predictors of uptake (starting at least 1 module) and engagement (watching at least 1 video). Those with higher levels of baseline distress (DQ5 score≥15) were more likely to start at least 1 module (B=2.18; *P*=.01) and watch at least 1 video (B=2.14, *P=*.007) compared with those with lower distress. In addition, those who were in a later year of their degree were more likely to engage (watch at least 1 video) than those in their first year (B=2.36; *P*=.008). Although it was not significant, there was a trend toward higher intervention uptake among those who received their trial condition allocation on time (within 3 days of completing the baseline assessment; B=1.78, *P*=.08). More detailed descriptive statistics for uptake and engagement with each intervention module are provided in Multimedia Appendix 2. The number of participants accessing each module declined over the order of modules. The most frequently accessed module was module 1 (145/243, 59.7%), and the least frequently accessed module was module 12 (83/243, 34.2%).

**Table 2:** Uptake and engagement with the Uni Virtual Clinic-Lite intervention.

Uptake and engagement			Values
**Participants who received the intervention (n=243), n (%)**			
	Started at least 1 module	168 (69.1)	
	Started all 12 modules	51 (21)	
**Participants who clicked through to the intervention modules (n=168), n (%)**			
	Started at least 1 video	114 (67.9)	
	Started all 12 videos	14 (8.3)	
	Watched ≥50% of at least 6 videos	42 (25)	
	Watched ≥50% of all 12 videos	11 (6.5)	
	Accessed at least 1 exercise	86 (51.2)	
	Accessed all 12 exercises	1 (0.6)	
	Accessed at least 1 quiz	151 (89.9)	
	Accessed all 7 quizzes	57 (33.9)	
Modules started, mean (SD)			5.10 (5.08)
Videos started, mean (SD)			3.58 (4.23)
Exercises accessed, mean (SD)			1.89 (3.02)
Quizzes completed, mean (SD)			4.14 (2.73)

### Primary Outcomes

Table 3 shows the estimated marginal means for the intervention and control groups on the primary outcomes at each measurement occasion. Significant decreases in depression (*F*_3,191.2_=4.3; *P*=.006) and generalized anxiety (*F*_3,192.1_=14.8; *P*≤.001) were observed over time in both trial groups, but no significant time-by-condition interactions were found, indicating that there was no significant difference in symptom reduction over time between conditions.

**Table 3:** Estimated marginal means for the intervention (Uni Virtual Clinic-Lite; UVC-Lite) and control groups at each measurement occasion and results of the mixed models analysis.

	Intervention (UVC-Lite) condition, mean (SE)					Control condition, mean (SE)					Occasion×condition	
	Baseline	Postintervention time point	3 months	6 months	Baseline		Postintervention time point	3 months	6 months	*F* test (*df*)		*P*
Depression^a^	9.7 (0.3)	8.9 (0.4)	9.1 (0.6)	9.1 (0.6)	9.0 (0.3)		8.1 (0.4)	7.9 (0.6)	8.9 (0.5)	0.51(3)		.68
Generalized anxiety^b^	7.8 (0.3)	6.7 (0.3)	7.6 (0.5)	8.1 (0.5)	7.3 (0.3)		5.9 (0.3)	6.6 (0.5)	7.4 (0.5)	0.22 (3)		.88
Social anxiety^c^	5.9 (0.2)	5.6 (0.3)	6.3 (0.4)	5.9 (0.4)	6.3 (0.2)		5.5 (0.3)	5.3 (0.4)	5.8 (0.3)	1.65 (3)		.18
Distress^d^	14.1 (0.2)	12.0 (0.3)	12.5 (0.5)	12.4 (0.5)	13.9 (0.2)		12.0 (0.3)	11.3 (0.5)	12.5 (0.5)	1.21 (3)		.31
Body appreciation^e^	3.4 (0.1)	3.4 (0.1)	3.6 (0.1)	3.4 (0.1)	3.3 (0.1)		3.3 (0.1)	3.4 (0.1)	3.4 (0.1)	0.38 (3)		.77
Quality of life^f^	28.6 (0.3)	28.5 (0.4)	28.1 (0.5)	28.8 (0.5)	28.5 (0.3)		28.8 (0.4)	29.3 (0.5)	28.9 (0.5)	1.23 (3)		.30
Well-being^g^	47.0 (1.1)	50.0 (1.5)	50.3 (2.2)	53.5 (1.9)	47.6 (1.1)		49.8 (1.5)	50.5 (2.2)	49.9 (1.8)	0.99 (3)		.40
Functioning^h^	45.2 (0.7)	46.4 (1.0)	46.3 (1.4)	46.9 (1.3)	45.6 (0.7)		47.3 (1.0)	47.5 (1.4)	45.4 (1.3)	1.01 (3)		.39
Self-efficacy^i^	19.1 (0.3)	19.1 (0.4)	19.2 (0.6)	19.2 (0.5)	18.9 (0.3)		19.1 (0.4)	19.5 (0.6)	19.7 (0.5)	0.47 (3)		.71
Academic self-efficacy^j^	34.5 (0.7)	36.1 (0.9)	36.1 (1.2)	37.3 (1.0)	34.0 (0.7)		35.2 (0.8)	37.0 (1.2)	35.9 (1.0)	0.73 (3)		.53

^a^Patient Health Questionnaire-9; range 0 to 27.

^b^Generalized Anxiety Disorder Scale-7; range 0 to 21.

^c^SAD: Social Anxiety Disorder Screener; range 0 to 16.

^d^Distress Questionnaire-5; range 5 to 25.

^e^Body Appreciation Scale-2; range 1 to 5.

^f^European Health Interview Surveys-8; range 0 to 32.

^g^5-item World Health Organization Well-Being Index; range 0 to 100.

^h^Recovering Quality of Life; range 0 to 80.

^i^General Self-Efficacy Scale-10; range 0 to 30.

^j^College Self-Efficacy Inventory; range 0 to 60.

### Secondary Outcomes

No significant differences were found between conditions over time on any secondary outcome variables (Table 3). However, both conditions showed significant reductions in distress (*F*_3,187.6_=25.0; *P*≤.001) and significant increases in body appreciation (*F*_3,173.5_=2.8; *P*=.04), quality of life (*F*_3,188.1_=4.3; *P*=.006), and academic self-efficacy (*F*_3,175.3_=5.6; *P*=.001) over time. Trial condition was not associated with help seeking from different sources at postintervention, 3-month follow-up, or 6-month follow-up.

### Analyses Among Engagers and Those With Higher Distress

No significant differences were found between conditions over time on any of the primary or secondary outcomes among those who engaged with the intervention (watched ≥1 videos). However, one time-by-condition interaction approached significance; nonsignificant reductions in distress were observed at the postintervention time point and 6-month follow-up among intervention group participants who watched ≥1 videos, compared with those in the control group (*F*_3,161.1_=2.3=; *P*=.08). No significant differences were found between conditions over time among those with higher levels of distress (DQ5 ≥15; n=260, 53.4%) at baseline.

### Satisfaction

Most participants who returned a postintervention survey reported being satisfied with the intervention; 81.3% (100/123) of the participants were either “very satisfied” or “somewhat satisfied” following the use of the UVC-Lite. Most participants (115/124, 92.7%) also reported that they would be willing to recommend the UVC-Lite to other students, and 75.6% (93/123) reported that they learned either “a lot” or “a fair bit” from the program. In terms of specific modules, 41.2% (47/114) of the participants reported that they liked the module on tackling negative and anxious thoughts the most, followed by the social anxiety module (45/114, 39.5%) and the study issues and procrastination module (44/114, 38.6%); a low proportion of students reported liking the module on thoughts of suicide (13/114, 11.4%) and social media and internet use (24/114, 21.1%).

## Discussion

### Principal Findings

This trial examined the effectiveness of a brief, video-based intervention for improving the symptoms of depression and generalized anxiety disorder among university students with moderate psychological distress. Brief, self-guided interventions delivered at scale have the potential to confer significant public health benefits, particularly in university-based settings where demand for resource-intensive services outstrips supply. However, in this trial, the intervention was not effective relative to the control condition on either of the primary outcomes or any of the secondary outcomes. This is at odds with the findings of systematic reviews and meta-analyses suggesting that internet-based programs can be effective for a range of mental health outcomes in university students [31-33]. Despite a degree of heterogeneity in digital interventions for university students in terms of the level of guidance, program length and mode of delivery, therapeutic approach, and program components, most digital interventions for students comprise text-based modules that are based on CBT. Reviews suggest that the effects generally tend to be higher in programs that are guided, are based on CBT, and target an indicated sample [30,32,33]. The results from the very few studies to date that have examined digital transdiagnostic interventions for university students are mixed. A recent randomized controlled trial of a guided digital transdiagnostic intervention was not found to be effective [7], whereas an older study comparing a transdiagnostic intervention to a waitlist control group found positive outcomes for anxiety and depression [36]. The findings of this trial, together with the limited and mixed evidence to date, suggest that uncertainty remains regarding the utility of digital transdiagnostic interventions for students or what components are needed in an effective intervention. More high-powered trials are needed, particularly those examining brief interventions and the use of video and other alternatives to text-based intervention modules.

There are several possible explanations for the null findings in this trial. First, engagement with the intervention was low. Approximately two-thirds of participants (168/243, 69.1%) accessed at least 1 component of the intervention, which is comparable to the rates of uptake in other studies of digital mental health interventions for university students (79.1%; range 32.2%-100%) [74]. However, among those who accessed the intervention, very few participants either completed these components (11/168, 6.5%) or accessed all the program modules (14/168, 8.3%), which is also consistent with other studies of digital interventions with students [33] and a study of a comparable transdiagnostic video-based intervention trialed in the general community [75]. Possible reasons for the low levels of engagement in this trial include low perceived need, administration issues with the trial, or lack of interest among participants in engaging with the trial materials. The trial was originally designed to recruit students with at least moderate levels of psychological distress at baseline (DQ5 score>8). However, due to concerns expressed by the university ethics review committee about the appropriateness of a low-intensity digital intervention for students with high levels of distress, eligibility criteria for the trial were revised to exclude students who screened positive for high levels of distress (DQ5 score>17). As indicated in the trial flow diagram, a significant number of students expressed interest in the trial but were deemed ineligible on the basis of high psychological distress (n=640). Students who were screened as eligible on the basis of moderate psychological distress (DQ5 score=8-17) scored within the mild ranges of the PHQ-9 and GAD-7 at baseline, possibly indicating a low perceived or actual need for the intervention. Higher symptom severity has been shown to be positively associated with engagement with digital interventions [76], and in this trial, those with higher baseline distress were more likely to engage with the intervention, supporting the theory that low rates of adherence were potentially driven by low perceived need.

Second, due to an administrative error, a proportion of trial participants (n=129, 26.5%) did not receive their condition allocation and materials within a timely window after registering for the trial and completing the baseline assessment. For some participants, there was a delay of almost 1 month. Although these errors were not significantly associated with negative impacts on intervention uptake or survey completion in this study, they nonetheless jeopardized the fidelity of the trial and may have somewhat contributed to the low intervention adherence and missing data. There is a risk that participants may lose interest in research participation if there are long gaps in receiving contact from the research team, which has led researchers to use strategies to maintain ongoing contact with research participants (email or phone reminders, newsletters, and social media groups). Randomization and initial email contact were conducted manually in this trial and were thus vulnerable to administrative error. Automating these procedures can help to minimize delays and errors in administering trial materials. Technical issues have been previously shown to be a major barrier to engagement with digital mental health interventions [76].

Third, the trial materials were delivered twice weekly to participants via email, which might have been missed or ignored by students who receive a large volume of email or check their email infrequently. Despite attempts to make these emails more engaging using eye-catching subject lines and wording, other methods for linking students to intervention materials should be considered, such as SMS text message reminders or prompts. Finally, it is possible that the intervention was not engaging for participants or that participants lacked interest in engaging with the trial materials. Young people may be changing in the ways that they engage with technology over time, such that an internet-based intervention may be less novel and attractive now than it was in the past. Moreover, the version of the intervention that was trialed in this study was neither co-designed with the students nor personalized or customizable. Lack of perceived relevance and personalization have been previously reported as the barriers to engagement with digital mental health interventions [76]. In this study, using co-design principles may have better aligned the intervention with the needs and expectations of the target users, leading to improved engagement.

The intervention was acceptable among the sample, as indicated by most participants indicating high satisfaction with the program and a willingness to recommend the program to others. Participants indicated that they liked the CBT-based modules the most (tackling negative and anxious thoughts and social anxiety), possibly because these modules contained tangible written exercises that users could complete or because these topics resonated the most with the participants in the sample. Engagement with the intervention was also higher among the later-year students than with the first-year students. This is noteworthy, given previous research suggesting that students may be at a heightened risk of developing mental health problems during their first year of university [77]. Possible delays in problem recognition or perceived need for treatment among students may account for the higher rates of engagement with the intervention among the later-year students in this trial. Students in their first year of university are often targeted with programs designed to help them adjust to university study and build social connections and study skills. Preventative mental health interventions could also be delivered at this time to assist students to build mental health literacy and skills in managing distress. However, findings from this trial suggest an ongoing need for accessible mental health interventions throughout a student’s university candidature.

### Strengths and Limitations

The study recruited a relatively larger sample than other trials of digital transdiagnostic interventions in university student populations [7,36]. The study also recruited a national sample from multiple universities, enhancing the generalizability of the findings. The study used a robust attention-control condition rather than a waitlist or no-treatment control. However, there are also several notable limitations, most of which are discussed in the previous section in relation to the study findings. In addition, there was low participation among male and gender-diverse students, limiting the generalizability of the findings to these groups. There was also high attrition in the trial, and although the data analytic methods used robustly accounted for missing data, the trial was likely underpowered due to not reaching the recruitment target and greater than expected dropouts from the assessments.

### Future Directions

The findings highlight several implications for both future research on the UVC-Lite intervention and the implementation of this and other digital interventions in higher education settings. UVC-Lite was designed to be a highly accessible and flexible intervention that does not require significant financial or technological resourcing to implement. It was associated with high satisfaction among students and was not associated with symptom deterioration. Given the challenges faced by universities in meeting demand for mental health services, interventions such as UVC-Lite have significant potential to assist students to manage low-level distress and prevent the development of severe symptomatology when delivered universally. However, low uptake and engagement among students with low levels of symptomatology is a significant challenge that requires further attention. Future studies should examine the effectiveness of the intervention in a more appropriately symptomatic sample. Moreover, very few studies have examined the drivers of engagement with digital interventions among university students [78] or robustly tested strategies to facilitate engagement. The order of delivery of the modules appeared to affect the engagement in the current trial, with earlier modules being accessed more frequently than later modules. This may suggest that 12 modules are too many or that tailoring modules to participant symptoms, rather than expecting participants to engage with all 12 modules may improve engagement. Delivery of the intervention was fully automated, with no human support or contact. Further studies are needed to test different implementation pathways for digital interventions in university settings, particularly those involving greater support (eg, through counseling centers or other health services or via the classroom). Longitudinal studies of mental health in university settings may provide useful data about critical time points for targeting digital interventions to different student groups. There is capacity for interventions such as UVC-Lite to be expanded and adapted to address a wider range of topics relevant to students from different ethnic and cultural backgrounds, and this should also be a focus of future research and implementation efforts.

### Conclusions

This study was the first to examine the effectiveness of a video-based transdiagnostic internet intervention for improving the mental health of university students. Although the intervention was designed to be highly engaging for a university student population, it was not effective in improving mental health outcomes, and sustained use of the intervention was low, possibly due to the low perceived need for the intervention among the study sample. Nonetheless, satisfaction with the intervention was high, and given the potential utility of brief transdiagnostic digital interventions in higher education settings, further research should examine the effectiveness of the UVC-Lite program among students with higher need for the program, as well as implementation pathways and strategies that optimize effective engagement with the program.

## Appendix

Multimedia Appendix 1 Screenshots of the Uni Virtual Clinic-Lite intervention.

Multimedia Appendix 2 Descriptive statistics for uptake and engagement with each intervention module.

Multimedia Appendix 3 CONSORT-EHEALTH checklist.

## Abbreviations

BAS-2: Body Appreciation Scale-2
CBT: cognitive behavioral therapy
CSEI: College Self-Efficacy Inventory
DQ5: Distress Questionnaire
EUROHIS-8: European Health Interview Survey-8
GAD-7: Generalized Anxiety Disorder Scale-7
PHQ-9: Patient Health Questionnaire-9
UVC-Lite: Uni Virtual Clinic-Lite
