# Supplementary material for: A Transdiagnostic Video-Based Internet Intervention (Uni Virtual Clinic-Lite) to Improve the Mental Health of University Students: Randomized Controlled Trial
Source: J Med Internet Res. 2024 Aug 13;26:e53598. doi: 10.2196/53598 (PMC11350308; doi:10.2196/53598)
Supplement: Multimedia Appendix 1 [file jmir_v26i1e53598_app1.docx]

Screenshots of the UVC-Lite intervention
