# Supplementary material for: A Transdiagnostic Video-Based Internet Intervention (Uni Virtual Clinic-Lite) to Improve the Mental Health of University Students: Randomized Controlled Trial
Source: J Med Internet Res. 2024 Aug 13;26:e53598. doi: 10.2196/53598 (PMC11350308; doi:10.2196/53598)
Supplement: Multimedia Appendix 2 [file jmir_v26i1e53598_app2.docx]

Descriptive statistics for uptake and engagement with UVC-Lite modules

| *N* = 243 | Mod 1 | Mod 2 | Mod 3 | Mod 4 | Mod 5 | Mod 6 | Mod 7 | Mod 8 | Mod 9 | Mod 10 | Mod 10 | Mod 12 |
| --- | --- | --- | --- | --- | --- | --- | --- | --- | --- | --- | --- | --- |
| Number started | 145 | 136 | 114 | 107 | 103 | 95 | 102 | 95 | 87 | 85 | 87 | 83 |
| Number started % | 59.7% | 56.0% | 46.9% | 44.0% | 42.4% | 39.1% | 42.0% | 39.1% | 35.8% | 35.0% | 35.8% | 34.2% |
| Number started video | 83 | 72 | 64 | 54 | 52 | 37 | 45 | 42 | 40 | 39 | 34 | 40 |
| Number started video % | 57.2% | 52.9% | 56.1% | 50.5% | 50.5% | 38.9% | 44.1% | 44.2% | 46.0% | 45.9% | 39.1% | 48.2% |
| Number watched 50% or more | 75 | 71 | 55 | 51 | 46 | 34 | 42 | 41 | 36 | 39 | 30 | 37 |
| Number watched 50% or more % | 90.4% | 98.6% | 85.9% | 94.4% | 88.5% | 91.9% | 93.3% | 97.6% | 90.0% | 100.0% | 88.2% | 92.5% |
| Number clicked pdf | 41 | 40 | 22 | 28 | 23 | 25 | 26 | 26 | 25 | 26 | 18 | 19 |
| Number clicked pdf % | 28.3% | 29.4% | 19.3% | 26.2% | 22.3% | 26.3% | 25.5% | 27.4% | 28.7% | 30.6% | 20.7% | 22.9% |
| Number completed quiz | 133 | N/A | 109 | 97 | 95 | N/A | 97 | 86 | N/A | N/A | 79 | N/A |
| Number completed quiz % | 91.7% | N/A | 95.6% | 90.7% | 92.2% | N/A | 95.1% | 90.5% | N/A | N/A | 90.8% | N/A |
